# Multiscale Structural Elucidation of Peptide Nanotubes by X-Ray Scattering Methods

**DOI:** 10.3389/fbioe.2021.654339

**Published:** 2021-03-29

**Authors:** Theyencheri Narayanan, Axel Rüter, Ulf Olsson

**Affiliations:** ^1^ESRF-The European Synchrotron, Grenoble, France; ^2^Division of Physical Chemistry, Lund University, Lund, Sweden

**Keywords:** peptide self-assembly, peptide nanotubes, peptide nanoribbons, X-ray scattering, SAXS

## Abstract

This mini-review presents the structural investigations of the self-assembled peptide nanotubes using X-ray scattering techniques. As compared to electron microscopy, scattering methods enable studies of nanotubes in solution under the appropriate physicochemical conditions and probe their formation mechanism. In addition, a combination of X-ray scattering methods allow the elucidation of structural organization from the molecular scale to the dimension of nanotubes.

## 1. Introduction

The aggregation of proteins can lead to a variety of complex structures, which are at the origin of many degenerative diseases (Ke et al., 2020). A well-known example is the fibrillar assemblies of amyloid peptides, formed by cleavage from a larger amyloid precursor protein, involved in neurodegenerative disorders (Serpell, 2000; Hamley, 2012). In the vast majority of cases, peptides self-assemble to fibrillar morphologies but under specific solvent and pH conditions, oligopeptides may form more organized nanotubes of nearly macroscopic dimension in length (Hartgerink et al., 1996; Valéry et al., 2011; Hamley, 2014). At the molecular level, both amyloid fibers and peptide nanotubes display the same characteristic feature, orthogonal antiparallel β-sheet structure, and sheet laminations (Mehta et al., 2008; Morris et al., 2013; Hamley, 2014). This unique structural signature shared by both pathogenic amyloid forming peptides and artificial functional peptides contrast their macroscopic properties, for example, the undesired effects of amyloid peptides in neurodegenerative diseases but beneficial roles of synthetic peptides in modern technologies (Ke et al., 2020). In particular, the self-assembly of synthetic biomimetic peptides enables a broader exploration of specific interactions and sequences than those possible with conventional amphiphilic molecules (Schneider et al., 2002; Zhao et al., 2013; Dasgupta and Das, 2019). As a result, relatively simple model peptide systems can be used to mimic more complex biological systems and processes (Ke et al., 2020; Levin et al., 2020).

The hierarchical self-assembly of short peptides to form uniform nanotubes of nearly macroscopic dimension has been the subject of numerous investigations (Valéry et al., 2011; Hamley, 2014). In specific cases due to the delicate interplay of hydrogen bonding, electrostatic and entropic interactions, the ribbon-like fibrillar structure curls and forms nanotubes of well-defined dimension. The wide range of polymorphism exhibited by peptide self-assemblies can be rationalized in terms of the free-energy landscape in which helical ribbons and nanotubes occupy closely located minima, with crystals at the lowest energy state (Adamcik and Mezzenga, 2018). Both helical ribbons and nanotubes have similar mean curvature and zero Gaussian curvature (Ke et al., 2020). The homogeneity in the diameter and limited tunability make them interesting candidates for templated growth of functional nanomaterials with potential applications in the biotechnology and nanotechnology (Reches and Gazit, 2003; Pouget et al., 2007; Valéry et al., 2011; Hamley, 2014; Levin et al., 2020).

This short review presents some of the recent studies of self-assembled peptide nanotube systems using X-ray scattering methods. In particular, small and wide angle X-ray scattering (SAXS and WAXS, respectively) elucidate the different hierarchical levels involved in the self-assembly process albeit lacking the chemical sensitivity (Narayanan et al., 2017). Indeed, the morphology of the peptide nanotubes is directly revealed by imaging methods such as cryogenic transmission electron microscopy (cryo-TEM) and atomic force microscopy (AFM) (Valéry et al., 2011; Hamley, 2014; Adamcik and Mezzenga, 2018), which is a decisive input for the quantitative X-ray modeling of the nanotubes in solution.

## 2. X-Ray Scattering Studies of Peptide Nanotube Self-Assembly

A prototypical case of the hierarchical self-assembly has been illustrated with the lanreotide octapeptide developed by Beaufour-Ipsen laboratory that forms a therapeutic gel (autogel®) (Valéry et al., 2003, 2011). At a lower lanreotide concentration than in the gel, monodisperse nanotubes of diameter about 25 nm are spontaneously formed. The supramolecular and molecular organizations within the tube wall were elucidated by SAXS and WAXS, and complementary techniques of electron microscopy and vibrational spectroscopies (Valéry et al., 2003). In this case, the exceptionally well-aligned fiber diffraction patterns revealed that the structural organization within the nanotubes wall is crystalline with low mosaicity, that in turn allowed constructing their electron density maps, which indicated a 2.07 nm alternation of aliphatic and aromatic residues that formed amyloid-β like fibers. The nanotubes are constituted by 26 of these fibers and consequently their diameter is very uniform. High-resolution SAXS further revealed the hexagonal packing of these nanotubes at high volume fractions (Valéry et al., 2003). The feasibility of serving these nanotubes as templates for biomineralization has been illustrated with a silica precursor resulting in double-walled silica nanotubes of uniform diameter and bundles of which assemble to centimeter-sized fibers (Pouget et al., 2007). Mutations in the peptide sequence can lead to different packing of aromatic residues, and modification of a single aromatic residue enabled a four-fold increase in nanotube diameter in the range of 10–36 nm (Tarabout et al., 2011). Further studies involving the effect of counterions revealed the specificity of anion size, demonstrating that counterions are not only regulating the charges on the surface of the nanotubes but also playing an important structural role, varying their diameter in the range of 19–26 nm (Gobeaux et al., 2012). By substituting monovalent by divalent counterions, double-walled nanotubes were observed and this morphological change has been attributed to competition between the adhesion force generated by divalent counterions and the mechanical stiffness of the peptide wall (Gobeaux et al., 2013).

Another nanotube forming peptide system investigated in detail from the structural perspective is the *CH*_3_*CO* − *KLVFFAE* − *NH*_2_, a sequence from the amyloid-β peptide, Aβ(16-22) (Lu et al., 2003; Childers et al., 2009). This short peptide self-assembles in acetonitrile/water solution at pH 2 into well-defined nanotubes with mean diameter about 52 nm and wall thickness 4.3 nm. Instead at pH 6, the peptide forms fibrils (Mehta et al., 2008). Using electron diffraction and complementary spectroscopic methods, the packing of peptides into bilayer leaflets within the tube wall was elucidated (Mehta et al., 2008, 2013). A model for the lamination of peptides involving antiparallel β-sheets and the curling of peptide bilayers to form nanotubes was proposed (Childers et al., 2009). Further work using peptides differing only in their N-terminal residue, phosphotyrosine vs. lysine, showed a coassembly as stacks of antiparallel β-sheets with precisely patterned charged lattices stabilizing the bilayer leaflet interface, creating nanotubes with dense negative external and positive internal surfaces (Li et al., 2016). Another sequence Aβ(13-22) with different N-terminal extensions enhanced the propensity for β-sheet laminations and transformed into nanotubes over a wider pH range (Liu et al., 2008).

The nanotubes formed by surfactant-like peptides are another case subjected to X-ray scattering investigations (Hamley, 2014). A well-studied system is the peptide without endcaps *AAAAAAK* (*A*_6_*K*) that self-assembles into nanotubes in water (Bucak et al., 2009). At higher concentrations, nanotubes orient to form a nematic-like phase. At even higher concentrations corresponding to a volume fraction in excess of 0.4, a lamellar phase has been reported (Cenker et al., 2011). The initial SAXS modeling suggested a wall thickness ≤ 1 nm and proposed a model involving the parallel packing of peptides in the nanotube walls (Castelletto et al., 2010). However, this model has been revised on the basis of other experimental evidences to that comprising bilayers of peptides perpendicular to the nanotube wall similar to that in the case of Aβ(16-22) (Hamley, 2014). Time evolution of SAXS intensity during the formation of nanotubes showed an exponential growth similar to a crystal growth process (Cenker et al., 2012). Further investigation of the homologous series *A*_*n*_*K* with *n* = 4, 6, 8, and 10 revealed that while *A*_6_*K* forms single-walled nanotubes in water above a threshold concentration (10%), *A*_4_*K* is fully soluble, and *A*_8_*K* and *A*_10_*K* assemble to rod-like aggregates even in dilute concentrations (Cenker et al., 2014). The latter structure has been revised to twisted ribbon with laminations of single stretched peptide molecules (Rüter et al., 2019) and a thermodynamic model has been presented for the transition between tube to ribbon structures (Rüter et al., 2020). The arginine-rich peptide R_3_L_12_ (arginine_3_-leucine_12_) forms a variety of nanostructures including nanotubes in aqueous solutions as a function of pH (Castelletto et al., 2020). These structures are supposedly built from α-helical antiparallel coiled-coil peptide dimers arranged perpendicular to the nanotube axis in a cross-α configuration.

## 3. Recent Examples of X-Ray Scattering Studies of Peptide Nanotubes

**Figure 1:**
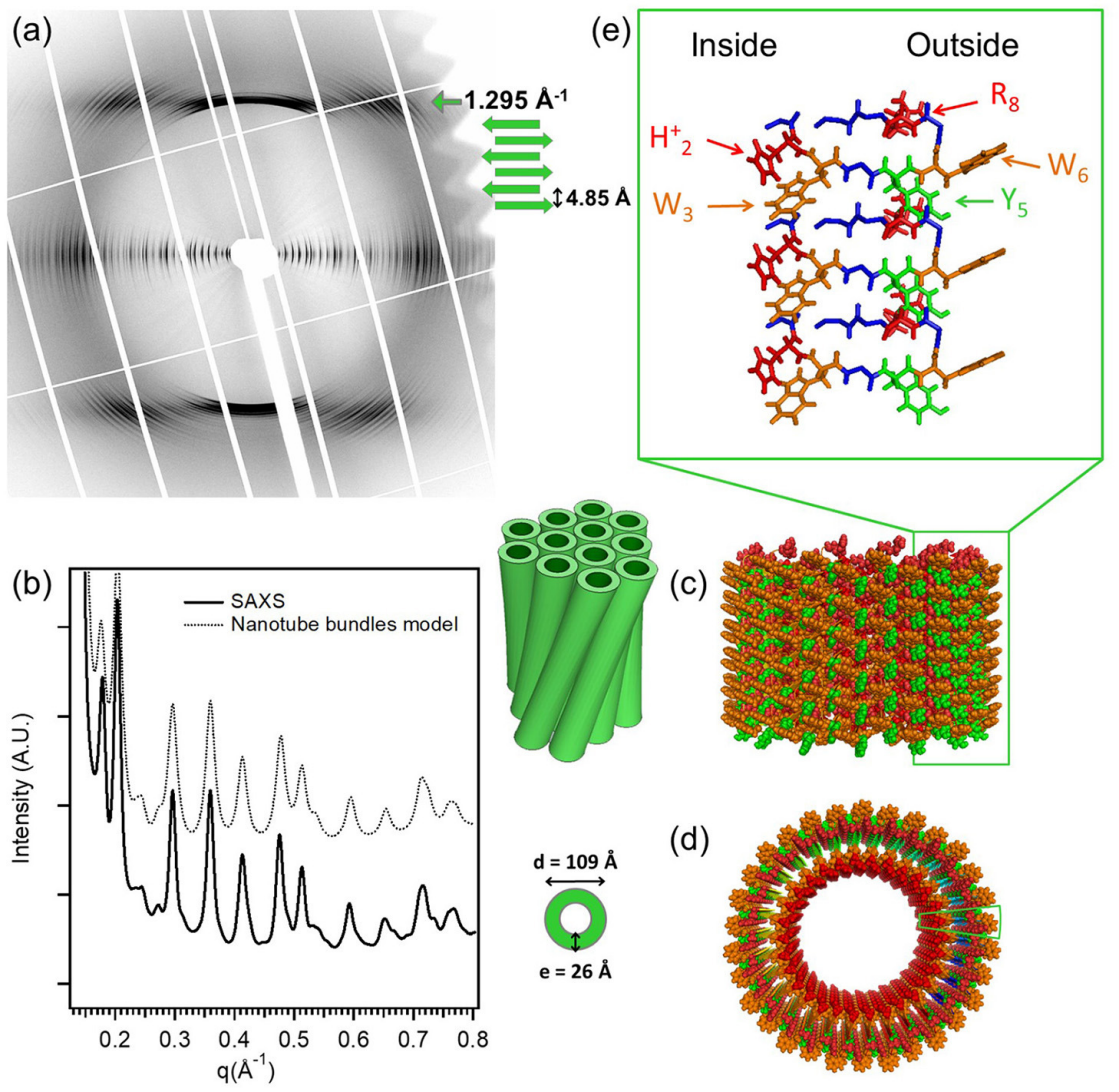
The molecular structure of the low-pH triptorelin nanotubes. Aligned **(a)** fiber diffraction pattern and **(b)** SAXS profile of bundles of small nanotubes. **(c)** Side and **(d)** top schematic views of the small nanotubes built from 30 protofilaments. **(e)** Molecular structure of the protofilaments forming the nanotube walls. Color code: W (orange), Y (green), protonated histidine (H_2_)^+^, and (R_8_)^+^ (red). The two green boxes in **(c,d)** underline the position of one protofilament that is enlarged in **(e)**. Reproduced from Valéry et al. (2015) licensed under a Creative Commons Attribution 4.0 International License.

A state-of-the-art example for X-ray diffraction investigation of peptide nanotube self-assembly has been presented by Valery et al. in the case of a decapeptide, triptorelin (Valéry et al., 2015). This peptide self-assembles to form monodisperse nanotubes (diameter ≃ 50 nm and wall thickness 2.6 nm) at higher pH (> 7.5). The organization within the nanotube wall is crystalline (2D monoclinic) as indicated by low mosaicity fiber diffraction patterns. These authors exploited a smart crystallization strategy to enable atomic resolution structural elucidation (Valéry et al., 2015). The conformational change of the peptide with pH was exploited to derive a deeper functional insight. At the atomic scale, it was revealed that the globular conformation at high pH is stabilized through a strong histidine-serine H-bond and a tight histidine-aromatic packing (Valéry et al., 2015). Lowering the pH induced histidine protonation, disrupting these interactions and triggering a large change to an extended β-sheet-based conformation. At lower pH (<6.5), triptorelin assemblies exhibit in the form of twisted nanotubes of smaller diameter (≃ 11 nm). High resolution small and wide angle X-ray diffraction was used to derive a molecular level structural model of the nanotube assembly. Figure 1 illustrates typical X-ray diffraction pattern from an oriented bundle of nanotubes and corresponding structural models at different hierarchical levels (Valéry et al., 2015). The well-defined meridional layerlines at 4.85 Å shows that the β-sheet H-bond network lies along the nanotube axis and forms protofilaments. The repeat distance along the nanotube axis is the size of the β-hairpin. The large number of Bragg peaks in the equatorial layer indicate the crystalline six-fold ordering of protofilments and 30 of which constitute a nanotube. The small-angle equatorial intensity is modeled in terms of hexagonal packing of nanotubes within the bundles. Furthermore, the nanotube wall thickness is about 2.6 nm, which is similar to that at higher pH but with an outer shell of 1 nm having an excess electron density than the inner layer. This corresponds to more aromatic residues located on the outer surface of the nanotube, which are likely involved in the close contact between nearest neighbors to form the twisted bundle.

**Figure 2:**
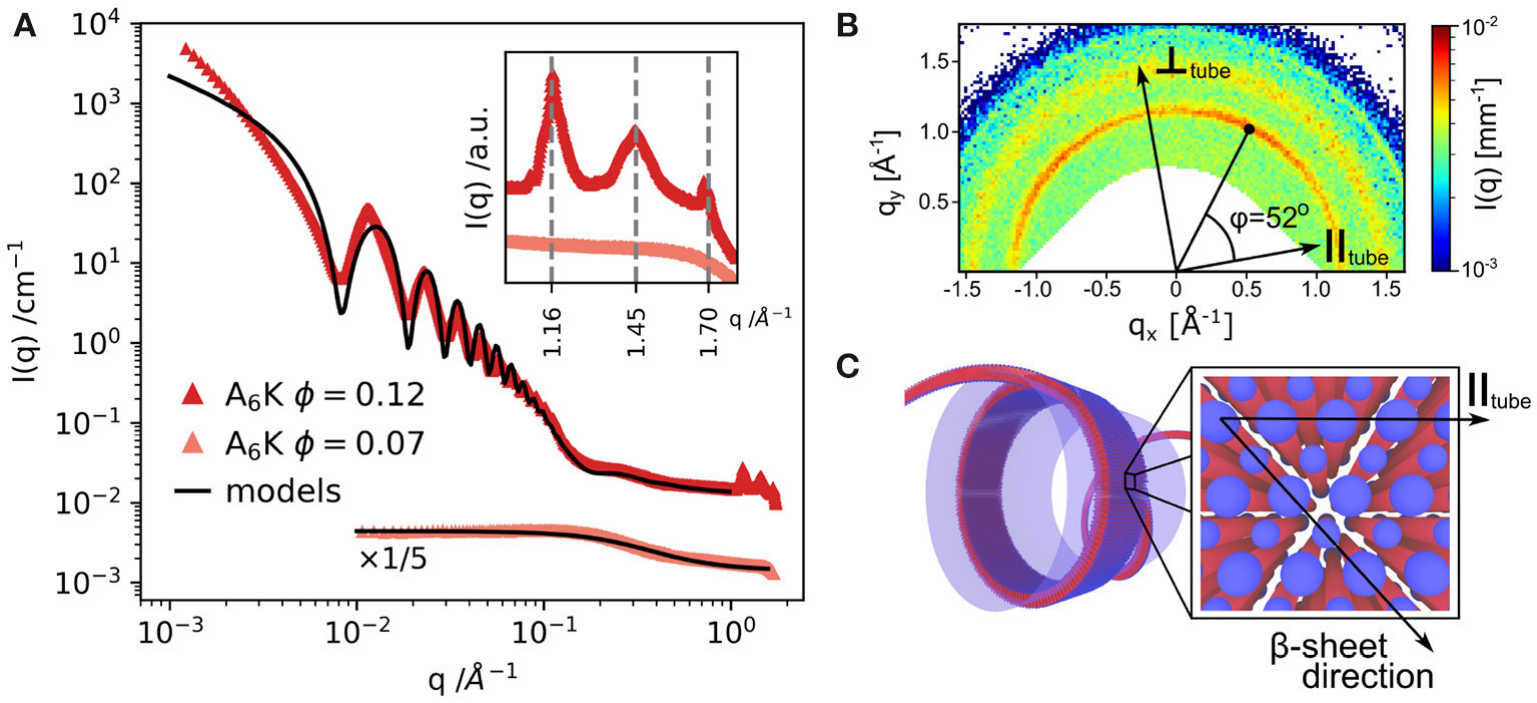
**(A)** SAXS and WAXS (inset) profiles from *A*_6_*K* peptide for two concentrations above and below the peptide solubility limit. **(B)** WAXS pattern from partially aligned nanotubes showing the peptide packing and helical pitch angle of 52^*o*^. **(C)** Model of the nanotube wall architecture: blue: hydrophilic peptide ends, red: hydrophobic core of the peptide sequence. Reproduced from Rüter et al. (2020) licensed under a Creative Commons Attribution 3.0 Unported License.

As mentioned before, in the *A*_*n*_*K* model peptide system the self-assembled structure is strongly dependent on the number of alanine residues in the chain, *n*, resulting in assemblies of tubes or twisted ribbons (Rüter et al., 2020). Figure 2A presents SAXS profiles of the nanotube forming *A*_6_*K* above and below the solubility limit (10%). The SAXS profile of dissolved peptides is well-described by the scattering function of Gaussian polymer coils with a radius of gyration of about 0.55 nm. At higher concentrations, the scattering profile shows the characteristic features from a tubular structure, which can be described by a hollow cylinder model with mean radius 30.8 nm, polydispersity 4% and wall thickness 3.3 nm. The deviations at lower q region is attributed to the packing of oriented tubes in domains. Figure 2B depicts the WAXS pattern from oriented nanotubes. The orientation of the peaks shows the helical pitch angle (52^*o*^) with β-sheets arranged on helical paths along the tube surface. The peaks in the WAXS pattern were assigned to a 2D oblique unit cell with the first peak at 1.16 nm^−1^ corresponding to the alanine stacking distance (Middleton et al., 2013). Considering the length of an extended peptide monomer, *l*_*p*_ = 2.5 nm and the tilt angle, the wall thickness of 3.3 nm may correspond to an interdigitated bilayer arrangement (Hamley, 2014). A similar bilayer architecture has been employed to model nanotubular morphologies formed by short peptides Aβ(16–22) (Mehta et al., 2013) and α*Sβ*1 (*NH*_2_ − *VLYVGSKT* − *COOH*) (Morris et al., 2013).

## 4. Other Systems Forming Self-Assembled Nanotubes

Not only short peptides but also a variety of amphiphilic molecules self-assemble to form nanotubes of comparable dimension. In many cases, SAXS has proven valuable for the elucidation of their structure and pathways of self-assembly in solution. An example is lithocholic bile acid in aqueous sodium or ammonium hydroxide solutions forming well-defined nanotubes of diameter in the range of 50 nm (Terech et al., 2006). In this case, SAXS revealed that the pathway of their formation is via helical ribbons. In concentrated suspensions, the oriented tubes self-organize to 2D hexagonal lattice with *p*6*m* symmetry (Terech et al., 2006). Using SAXS, cryo-TEM, and AFM, derivatives of cholic acid (Travaglini et al., 2013) and sodium lithocholate (Gubitosi et al., 2014) have been shown to self-assemble into nanotubes. Furthermore, in mixtures of sodium lithocholate and its mannose derivative, the nanotube radius could be tuned by the mixing ratio (Gubitosi et al., 2015). Certain amino acid amphiphiles, like N-α-lauryl-lysyl-aminolauryl-lysyl-amide, have also been shown to form nanotubes in solution (Ziserman et al., 2011). Here, nanotubes were observed to form slowly, over a period of several weeks. Following the process using cryo-TEM, it was demonstrated how nanotubes evolved from originally thin fibers via twisted and helical ribbons (Ziserman et al., 2011). Besides, some sterols and γ-oryzanol in edible oils form nanotubes with diameters ranging from 6 to 8 nm (Bot et al., 2009) and the network of these tubes resulting in an organogel. Another case is the self-assembly of DNA minor groove-binding heterocyclic ligand DB921 in aqueous solutions containing chloride or bromide salts (Mizuta et al., 2018). The single-walled nanotubes of diameters 26–32 nm are developed from helical ribbons which close to form tubes only in the presence of chloride or bromide counterions.

Amphiphilic diblock copolypeptoids having domains chemically distinct and congruent in size and shape self-assemble in water into nanotubes with crystalline walls (Sun et al., 2016). These peptoids are like molecular tiles and their length determines the nanotube diameter (4–10 nm). The nanotube wall is composed of stacked, porous crystalline rings, which are held together primarily by side-chain van der Waals interactions. Bolaamphiphiles consisting of a sugar residue, an alkyl chain, and an amino group (NKNT2-C18) are another type of molecules exhibiting propensity to self-assemble and form nanotubes in aqueous solution with diameters in the range of 18–20 nm (Takahashi et al., 2019). In this case, the formation mechanism is not via helical ribbons but more like by closure of the well-organized monolayer membranes. Finally, the programmable route to design nanotubes of tailored diameter and desired chirality is via single-stranded DNA bricks strategy (Sun et al., 2019). In this case, the width of DNA helical tubes is controlled by the rigidity and curvature of repeating units through their thickness and helical twist density, respectively. Nanotubes, as well as the twisted or helical ribbons, that form in these self-assembling systems, are considered to be consequences of molecular chirality (Helfrich and Prost, 1988; Chung et al., 1993; Selinger et al., 2001). However, achiral gemini-tartrate amphiphiles complexed with chiral tatrate anions form ribbons and tubes due to induced chirality at a supramolecular level (Brizard et al., 2007). It has been possible to resolve the molecular structure of such self-assembled systems and correlate to the macroscopic properties (Oda et al., 2008).

## 5. Summary

This mini review presented a brief overview of X-ray scattering investigations of self-assembled peptide nanotube systems. The key advantage of X-ray scattering method is that the structural information can be elucidated in real solvent and under appropriate thermodynamic conditions, over a broad range of concentrations. In addition, external perturbations such as temperature (Cenker et al., 2011), shear flow (Narayanan et al., 2020), and electric or magnetic fields (Pandey et al., 2017) can easily be imposed. A combination of SAXS and WAXS methods enable hierarchical structural elucidation from the crystalline molecular packing up to the micron scale dimension of the nanotubes. In addition, the coexistence of different structural moieties can be identified on a quantitative scale (Narayanan et al., 2021). Complementary real space information from cryo-TEM and AFM provide decisive input for constraining the solution structural model. Although, both cryo-TEM (Ziserman et al., 2011) and frequency modulation AFM (Sugihara et al., 2013) are capable of revealing subnanometer molecular structure of nanotubes, X-ray modeling yields more quantitative structural parameters averaged over a large ensemble.

High brilliance SAXS and WAXS methods enable deciphering of weak structural features superimposed on a large background, which could be useful in studies involving self-assembled functional peptide systems. One potential avenue is exploring the complex pathways of self-assembly involving the coexistence of multiple structural motifs. A particular challenge is formulating theoretical models that can predict structural parameters from molecular properties (Nyrkova and Semenov, 2010; Zhang et al., 2019). The analysis software tools need to be better optimized for complete modeling of the self-assembled structures from the known molecular architecture.

## Author Contributions

TN wrote the first draft of the manuscript. All authors contributed in the subsequent revisions of the manuscript.

## Conflict of Interest

The authors declare that the research was conducted in the absence of any commercial or financial relationships that could be construed as a potential conflict of interest.
